# Implementation of Artificial Intelligence-Assisted Endoscopy Across Canada—The CAG Artificial Intelligence Special Interest Group

**DOI:** 10.1093/jcag/gwac030

**Published:** 2022-10-17

**Authors:** Daniel von Renteln, Michael F Byrne, Clarence Wong, Charles Menard, Fergal Donnellan, Alan Barkun

**Affiliations:** Division of Gastroenterology, University of Montreal Hospital Research Center (CRCHUM) and Medical Center (CHUM), Montreal, Quebec, Canada; Division of Gastroenterology, Vancouver General Hospital and University of British Columbia, Vancouver, British Columbia, Canada; Division of Gastroenterology, Royal Alexandra Hospital, University of Alberta, Edmonton, Alberta, Canada; Division of Gastroenterology, University of Sherbrooke, Sherbrooke, Quebec, Canada; Division of Gastroenterology, Vancouver General Hospital and University of British Columbia, Vancouver, British Columbia, Canada; Division of Gastroenterology and Hepatology, McGill University Health Centre, McGill University, Montreal, Quebec, Canada

Artificial intelligence (AI) solutions use deep neural networks that imitate human brain neural interconnections. By doing so AI solutions can analyze endoscopy videos or images during or after an endoscopy (1). This allows feedback information to be superimposed on the endoscopy screen, assisting endoscopists, for example, during colonoscopy with the detection and differentiation of polyps, and prediction of histology or disease activity such as in inflammatory bowel disease. There are many other possible endoscopic applications.

AI is expected to create many disruptive technologies in endoscopy with far-reaching and significant impact on quality and practice in general. Since endoscopy relies heavily on pattern recognition within video or image frames, AI seems an ideal technology to assist endoscopists in reaching quality benchmarks by providing live feedback. Colonoscopy quality metrics such as adenoma detection rate (ADR), withdrawal time of the colonoscope, cecal intubation rate and bowel cleanliness score are all ideal targets for automatic assessment and documentation using adapted AI solutions (1,2). Since these quality metrics are strongly linked to favorable patient outcomes, a positive clinical impact attributable to AI is to be expected.

The first wave of AI applications that has been implemented in endoscopy units includes automated and integrated AI solutions to assist endoscopists in the detection and classification of colorectal polyps. Many image-based modalities have been developed to increase ADR, since an increase in ADR is closely linked to the prevention of interval colorectal cancers (CRC) (3). Although most image-based technologies have shown no or only moderate improvement in ADR, AI-based solutions have been shown to increase ADR by more than 10% (4) in robust international randomized controlled trials (RCTs). Table 1 summarizes the more than 10 available RCTs demonstrating an increase in ADR. This illustrates the tremendous potential of AI to impact the most important quality metric of endoscopic CRC prevention. Furthermore, the increase in ADR is independent of patient selection—that is, the benefits in increased ADR exist whether implemented for patients at average risk screening or following a positive fecal immunochemical test. More importantly, the increase in ADR is observed when using AI for both trainees as well as experienced endoscopists (5). Since an increase in ADR is closely linked to a reduction in interval cancer risk, the relevance of these findings cannot be overstated, and rapid integration of AI-based systems into clinical practice seems important. Meanwhile, almost all endoscopy platform providers offer integrated solutions for polyp detection, and platform-independent solutions are also now available, some having been approved by Health Canada.

**Table 1.** Results of adenoma detection from randomized controlled trials using AI

Author	Year	Intervention	Control	Population	Male	ADR	OR/ RR
Wang	2019	CADe (real-time automatic polyp detection system (Shanghai Wision AI Co., Ltd.)	Routine colonoscopy	522 (CADe)	50.4% (CADe)	29.1% (CADe)	N/A
				536 (routine colonoscopy)	46.5% (routine colonoscopy)	20.3% (routine colonoscopy	
Wang	2020	CADe(EndoScreener; Wision AI, Shanghai, China)	Conventional white-light colonoscopy (WL)	484 (CADe)	50% (CADe)	34% (CADe)	OR 1.36; 95% CI 1.03 – 1.79
				478 (conventional white-light colonoscopy)	53% (WL)	28% (WL)	
Repici	2020	CADe(Convolutional Neural Network (GI-Genius; Medtronic)	Routine colonoscopy	341 (CADe)	50.4% (CADe)	54.8% (CADe)	RR 1.30; 95% CI 1.14 – 1.145
				344 (routine colonoscopy)	49.6% (routine colonoscopy)	40.4% (routine colonoscopy)	
Liu	2020	CADe(Henan Xuanweitang Medical Information Technology Co., LTD., Zhengzhou City, Henan Province, China)	Routine colonoscopy	508 (CADe)	51.9% (CADe)	39.1% (CADe)	OR 1.637; 95% CI 1.201 – 2.220
				518 (routine colonoscopy)	55.4% (routine colonoscopy)	23.8% (routine colonoscopy)	
Gong	2020	CADe(ENDOANGEL)	Routine colonoscopy	355 (CADe)	53% (CADe)	16%(CADe – ITT)	OR 2.30; 95% CI 1.40 – 3.77 (ITT) OR 2.18; 95% CI 1.31 – 3.62 (PPA)
				349 (routine colonoscopy)	45% (routine colonoscopy)	8%(routine colonoscopy – ITT)	
						17% (CADe – PPA)	
						8% (routine colonoscopy – PPA)	
Repici	2022	CADe(GI Genius, Medtronic)	High-definition colonoscopy (HD)	330 (CADe)	52.7% (CADe)	53.3% (CADe)	RR 1.22; 95% CI 1.04 – 1.40
				330 (HD)	47.3% (HD)	44.5% (HD)	
Quan	2022	CADe(ndoVigilant Inc, Mar- yland, United States)	High-definition colonoscopy (HD)	300 (CADe)	55% (CADe)	43.7% (CADe for screening)	N/A
				300 (HD)	57% (HD)	37.8% (HD for screening)	
						66.7% (CADe for surveillance)	
						59.7% (HD for surveillance)	
Rondonotti	2022	CADe (CADEYE Fujifilm Co., Tokyo, Japan)	High-definition white light colonoscopy (HDWL)	405 (CADe)	52.6% (CADe)	53.6% (CADe)	RR 1.18; 95% CI 1.026 – 1.361
				395 (HDWL)	49.6% (HDWL)	45.3% (HDWL)	

ADR, adenoma detection rate; AI, artificial intelligence; CADe, computer aided polyp detection; HDWL, high defnition white light; ITT, intention to treat; OR, odds ratio; PPA, per protocol analysis; RR, relative risk; WL, white light.

AI can characterize polyps and predict histology with high accuracy. While experienced endoscopists are usually able to predict pathology without needing AI, AI solutions can increase diagnostic accuracy for less experienced endoscopists (6). Studies have shown that when using AI, all endoscopists can meet critical accuracy benchmarks independent of their optical diagnosis skills, and perhaps sufficient for adopting a resect and discard colon polyp strategy. This will pave the way to replacing pathology for diminutive polyps with optical diagnosis, making colonoscopy practice more cost-effective (7). However, distinction between serrated or high-grade dysplastic pathology from other neoplastic polyps is, at present, not possible using AI. Future AI solutions will need to be trained to recognize this important granularity of polyp pathology subtypes (8). Once this is possible, widespread implementation of resect and discard and/or diagnose and leave strategies can be expected.

Other AI solutions improve quality monitoring with automated assessment of quality metrics (such as bowel preparation, and completeness of the exam). For upper endoscopy, AI can assist in the assessment of the extent of Barrett’s esophagus, and in identifying areas with high-grade dysplasia or cancer (9). To improve productivity, workflow and quality control in endoscopy units, AI can create automated reports or automated analyses of databases, such as institutional or individual ADR (10). Development, research and regulatory approval have all allowed rapid progress in the development and dissemination of AI solutions.

To further develop and promote AI technology, we have formed a special interest group (SIG) in AI at the Canadian Association of Gastroenterology (CAG. This CAG AI SIG core group is currently comprised of six gastroenterologists (the authors of this opinion piced) from five Canadian institutions across three provinces. We have started evaluating AI technologies using cohort studies and randomized controlled trials, and are in the process of establishing video and data biobanks to accrue raw data from which additional novel AI solutions can be created. Our research activities to date have been supported through seed funding from CAG, and we have organized and hosted webinars and sessions at Canadian Digestive Diseases Week (CDDW), inviting international experts on selected pertinent topics. Further activities of group members include the development and implementation of AI curricula since the next generation of gastroenterologists needs to be trained to develop and implement AI solutions at institutions across Canada. The CAG AI SIG has an open model inviting new members, industry and AI researchers to maximize the potential that this novel technology offers in improving endoscopy quality and patient outcomes.

## References

[1] Taghiakbari M , Mori Y, von Renteln D. Artificial intelligence-assisted colonoscopy: A review of current state of practice and research. World J Gastroenterol. 2021;27:8103–22. 35068857 10.3748/wjg.v27.i47.8103 PMC8704267

[2] Lee JY , Calderwood AH, Karnes W, Requa J, Jacobson BC, Wallace MB. Artificial intelligence for the assessment of bowel preparation. Gastrointest Endosc. 2022;95:512–8.e1. 34896100 10.1016/j.gie.2021.11.041

[3] Corley DA , Jensen CD, Marks AR, et al. Adenoma detection rate and risk of colorectal cancer and death. N Engl J Med. 2014;370:1298–306. 24693890 10.1056/NEJMoa1309086 PMC4036494

[4] Spadaccini M , Iannone A, Maselli R, et al. Computer-aided detection versus advanced imaging for detection of colorectal neoplasia: A systematic review and network meta-analysis. Lancet Gastroenterol Hepatol. 2021;6:793–802. 34363763 10.1016/S2468-1253(21)00215-6

[5] Repici A , Spadaccini M, Antonelli G, et al. Artificial intelligence and colonoscopy experience: Lessons from two randomised trials. Gut. 2022;71:757–65. 34187845 10.1136/gutjnl-2021-324471

[6] Rondonotti E , Hassan C, Tamanini G, et al. Artificial intelligence-assisted optical diagnosis for the resect-and-discard strategy in clinical practice: The Artificial intelligence BLI Characterization (ABC) study. Endoscopy 2022. 10.1055/a-1852-0330 35562098

[7] Zarandi-Nowroozi M , Djinbachian R, von Renteln D. Polypectomy for diminutive and small colorectal polyps. Gastrointest Endosc Clin N Am. 2022;32:241–57. 35361334 10.1016/j.giec.2021.12.009

[8] Taghiakbari M , Pohl H, Djinbachian R, et al. What size cutoff level should be used to implement optical polyp diagnosis? Endoscopy 2022. 10.1055/a-1843-9535 35668663

[9] Spadaccini M , Vespa E, Chandrasekar VT, et al. Advanced imaging and artificial intelligence for Barrett’s esophagus: What we should and soon will do. World J Gastroenterol. 2022;28:1113–22. 35431503 10.3748/wjg.v28.i11.1113 PMC8985480

[10] Tinmouth J , Swain Mhi D, Chorneyko K, et al. Validation of a natural language processing algorithm to identify adenomas and measure adenoma detection rates across a health system: A population-level study. Gastrointest Endosc. 2022. 10.1016/j.gie.2022.07.009 35843286

